# Recent Progress in Antibacterial Surfaces for Implant Catheters

**DOI:** 10.34133/bmef.0063

**Published:** 2025-02-13

**Authors:** Jia Hu, Qing Yu, Lei Wang, Hengchong Shi, Shifang Luan

**Affiliations:** ^1^ State Key Laboratory of Polymer Physics and Chemistry, Changchun Institute of Applied Chemistry, Chinese Academy of Sciences, Changchun 130022, P. R. China.; ^2^ School of Applied Chemistry and Engineering, University of Science and Technology of China, Hefei 230026, P. R. China.

## Abstract

Catheter-related infections (CRIs) caused by hospital-acquired microbial infections lead to the failure of treatment and the increase of mortality and morbidity. Surface modifications of the implant catheters have been demonstrated to be effective approaches to improve and largely reduce the bacterial colonization and related complications. In this work, we focus on the last 5-year progress in the surface modifications of biomedical catheters to prevent CRIs. Their antibacterial strategies used for surface modifications are further divided into 5 classifications through the antimicrobial mechanisms, including active surfaces, passive surfaces, active and passive combination surfaces, stimulus-type response surfaces, and other types. Each feature and the latest advances in these abovementioned antibacterial surfaces of implant catheters are highlighted. Finally, these confronting challenges and future prospects are discussed for the antibacterial modifications of implant catheters.

## Introduction

Catheter-related infections (CRIs) are a great threat to human health and also cause huge financial expenditures. It has been reported that 98% of urinary tract infections are associated with catheters, and they cost about $2.5 billion a year in the United States [1–3]. Likewise, catheter-associated bloodstream infections are a common hospital-acquired illness. Central venous catheters (CVCs) are regarded as one of the most important medical devices, yet their use is often relevant to serious complications, such as infections and thrombosis [4,5]. It has been reported that more than 80,000 of CVC-associated complications with a death rate of 12 to 25% occurred in intensive care units [6]. Moreover, the risk of co-occurring of these complications will largely increase especially in the long-term indwelling of catheters. Recently, Teja et al. [7] count the incidence of 15 complications associated with CVC placement, and the obtained results indicate that the rate of severe complications including arterial intubation, pneumothorax, infection, or deep vein thrombosis is approximately 3% after a CVC placed for 3 d. Some side effects have also been observed in the case of used peripheral versus catheters, for example, phlebitis, partial dislodgement, and infiltration [8].

Multitudinous researches have indicated that the medical device surfaces are closely related to the process of bacterial infections. Medical catheters are usually made of polymer materials, such as silicone, polyurethane, and latex, which may allow bacteria and fungi to thrive. In fact, the main complications arise from the ability of microbes to attach to surfaces and the formation of biofilms, which can protect microbes and assists them in persisting in the host [3,9]. Additionally, biofilms serve as a barrier against antimicrobials [10,11]. The formation of biofilm is closely ascribed to CRIs. Taking the CVC-associated complications mentioned above as example, the clinical methods to address these issues are administrating anticoagulants and antibiotics. Serious adverse reactions may be raised for their excessive use, including hypersensitivity and heparin (Hep)-induced thrombocytopenia, as well as the emergence of antibiotic-resistant bacteria [12,13]. Therefore, it is of great importance to design a bioactive catheter with many functions to inhibit bacterial colonization and reduce related complications.

In the last few decades, the developments of antimicrobial surfaces and biofilm-caused infections have brought about widespread concerns in the field of implantable devices. Several new strategies have been developed to replace the traditional use of antibiotics. Some scholars make review from the antibacterial surface design method (physical or chemical methods) and special material antibacterial application [14–17]; others review from the perspective of different functions such as antifouling, sterilization, and blood compatibility [18–21]. In 2020, our group published a review in the surface modification of catheters and also the test methods used to evaluate the performances of modified catheters [22]. At present, the antibacterial materials with a smart response have been designed to monitor and track early bacterial infection, and thus, the risk of infection can be reduced by implementing timely interventions [23,24]. Some reviews of the type of materials with this capacity have been published [25,26]. But few comprehensive works focus on the surface modifications of implant catheters and understanding the different mechanisms. In this work, we summarized the last 5-year progress on the surface antimicrobial strategies of medical antibacterial catheters. These corresponding progresses have been divided into 5 sections based on the antimicrobial mechanisms, and we summarized their merit and demerit. At last, these evaluation results and future trends are given with the aim of inspiring researchers to explore much better antimicrobial strategies.

## Typical Strategies for Constructing Antibacterial Surfaces on Medical Catheters

**Fig. 1.**
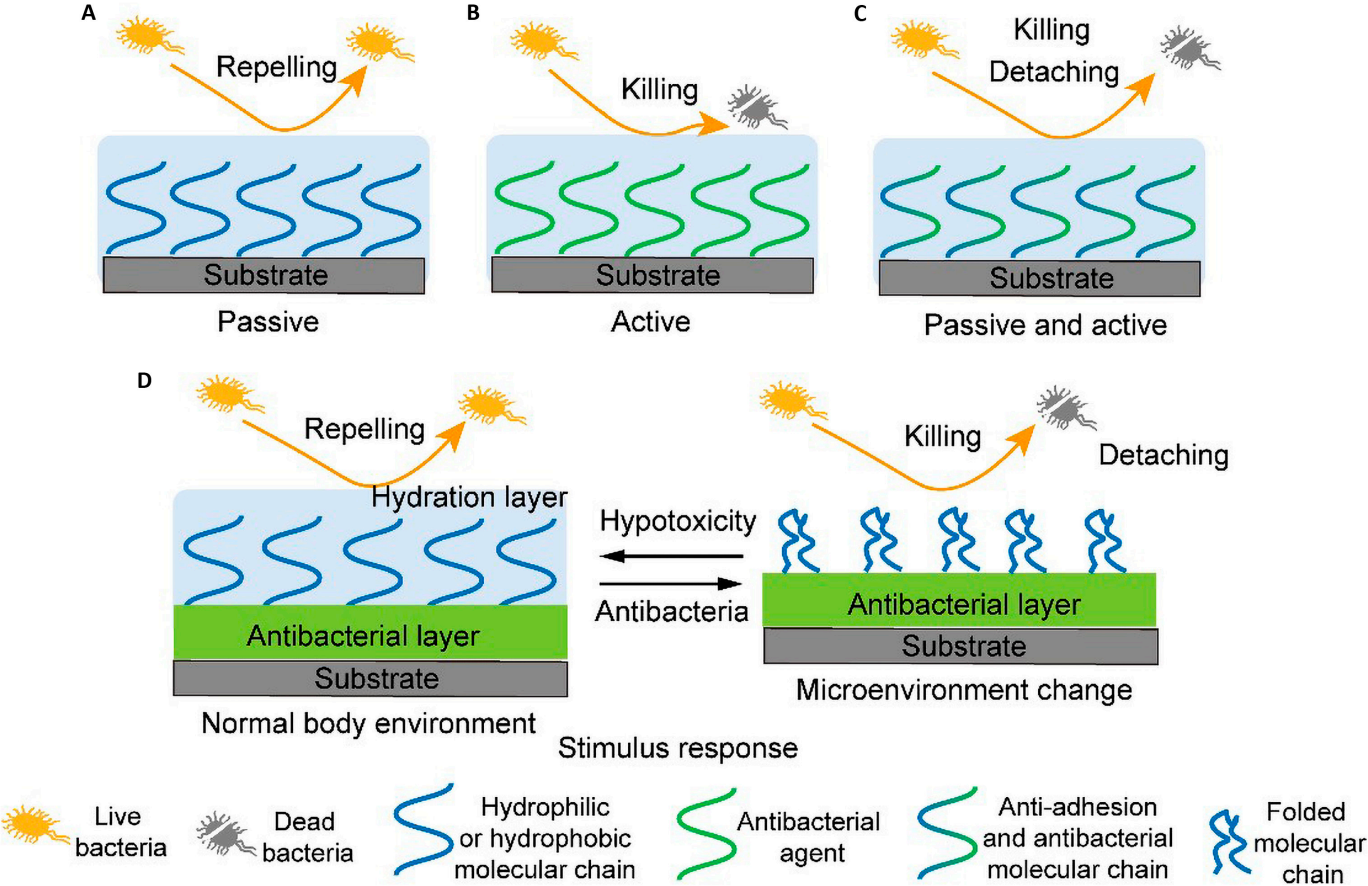
Schematic representation of typical strategies for constructing antibacterial surfaces on medical catheters. (A) Passive strategy. (B) Active strategy. (C) Passive and active strategy. (D) Stimulus response.

Proteins such as fibronectin and fibrin in thrombus are conducive to bacterial adhesion and promote the formation of biofilm. In turn, the presence of these biofilms can induce platelet aggregation and exacerbate thrombus accumulation [27,28]. Therefore, endowing catheters with enhanced anticoagulant and antibacterial properties is necessary. Many studies have been used to replace the clinical use of anticoagulants and antibiotics by modifying the surface of catheter. According to the different mechanisms, these antibacterial catheters can be divided into 5 main types: passive, active, active–passive combined, stimulus-responsive, and other types (Fig. 1).

Passive antibacterial catheters prevent the adhesion of bacteria or clots by constructing superhydrophilic or superhydrophobic surfaces (Fig. 1A). Hydrophilic materials can be introduced to enhance the hydrophilicity of solid surfaces, such as polyethylene glycol (PEG), polyvinylpyrrolidone (PVP) [29], zwitterionic polymer [30–32], and polyacrylic acid (PAA). They can form a tight hydrated layer to prevent nonspecific adsorption by bacteria. For example, a benzophenone-derived antibacterial agent with zwitterion structure can form a hydration layer to prevent early bacterial adhesion, and it can kill bacteria with high sensitivity under the existing alkaline phosphatase within infection sections [30]. Moreover, the superhydrophobic surface has a low surface energy, and the superhydrophobic coating can increase the contact angle between bacteria and liquid to decrease the bacterial adhesion sites [33]. For example, fluoropolymers can effectively prevent the risk of bacterial adhesion due to their low surface energy. Meanwhile, they maintain good cellular compatibility, and this is very important for biomedical applications [34].

But when dealing with complex pollutants, these antifouling methods have some characteristic defects. These abovementioned passive surfaces are sometimes not effective in preventing the growth, proliferation, and colonization of bacteria and the formation of biofilms after a long period of operation. On the contrary, active attacks on antibacterial surfaces made from antibiotics, fungicides, nitric oxide, and ion-released antibacterial metals can directly kill attached bacteria (Fig. 1B). These molecules are applied to biomaterials by physical adsorption, immersion in polymer substrates, or complexation. For instance, our research team prepared a water-insoluble antibacterial coating by utilizing the electrostatic interaction between anion surfactant, 1,4-bis(2-ethylhexyl) sodium sulfosuccinate, and cation ε-poly-ʟ-lysine [35]. What is more, some special structures can be used as fungicide carriers to enhance the antibacterial effect, for example, biomimetic rough nano-silica can adhere pathogens specifically to strengthen sterilizing effect, and some multilayer structures can release antimicrobials in response, decreasing cytotoxicity [36]. After implantation, the fungicide moves onto the attached microbial cells and is released in high local concentrations to inhibit initial surface colonization and prevent biofilm formation. This approach allows high doses of antimicrobial agents to be released without exceeding the toxicity threshold, thus reducing the development of resistance. However, the disadvantage of this technique is that the release is uncontrolled and lacks long-term characteristics [37]. Nonrelease methods are those based on coated materials that are in principle capable of counteracting bacterial adhesion and biofilm formation when microorganisms come into contact with the coated surface. Most of these coatings are based on polymers with antimicrobial activity such as cationic polymers and antimicrobial peptides; some specific examples are imidazole ionic liquid (IL)-typed cationic antimicrobials [38] and poly (γ-glutamic acid) (γ-PGA) [39]. This strategy has long antibacterial activity and low toxicity [40]. Some compounds, such as quaternary ammonium compounds and polypeptides, have been evaluated as promising contact killers. However, the main problem with this strategy is that bioactive surfaces may be deactivated when coated with proteins in body fluids [40].

As shown in Fig. 1C, the combination of active and passive materials is considered an “ideal” antibacterial coating that prevents the initial bacteria from adhering and also killing bacteria near the surfaces. They can be prepared during the coating process by blending or covalently linking antifouling and antibacterial polymers. For example, the cationic polymer is cytotoxic, so our team designed a coating with bactericidal effect under dry conditions and anti-adhesion in the water environment by photografting a zwitterionic hydration layer on the outside of the cationic bactericidal layer, and the coating has better biocompatibility than the naked cationic references [41].

The infections of the implant sites are usually not detected in time, and this delayed perception is likely to cause irreversible pathological damage and cognitive and behavioral abnormalities, and seriously, it may lead to increased mortality [42,43]. Therefore, it is of great significance to develop a coating with tracking and monitoring functions. Bacterial infection can cause changes in the microenvironment, such as temperature rise, pH change [44], the release of relevant enzymes, and glutathione concentration changes. These changes have been shown to enable to design intelligently responsive coating materials (Fig. 1D). For example, photodynamic therapy (PDT) [45–48] and photothermal therapy (PTT) [49] can control the release of reactive oxygen species (ROS) to achieve bactericidal effects. The ROS, such as hydroxyl free radical, superoxide free radicals, and hydrogen peroxide, can break down the active substances in bacterial cells and stimulate the oxidative stress [50]. Lately, catheters with anticoagulation and location drug release properties have been shown to be prepared using liquid gating techniques.

## Recent Advances in Constructions of Antibacterial Surfaces for Medical Catheters

### Passive strategy for constructing antibacterial surfaces

**Fig. 2.**
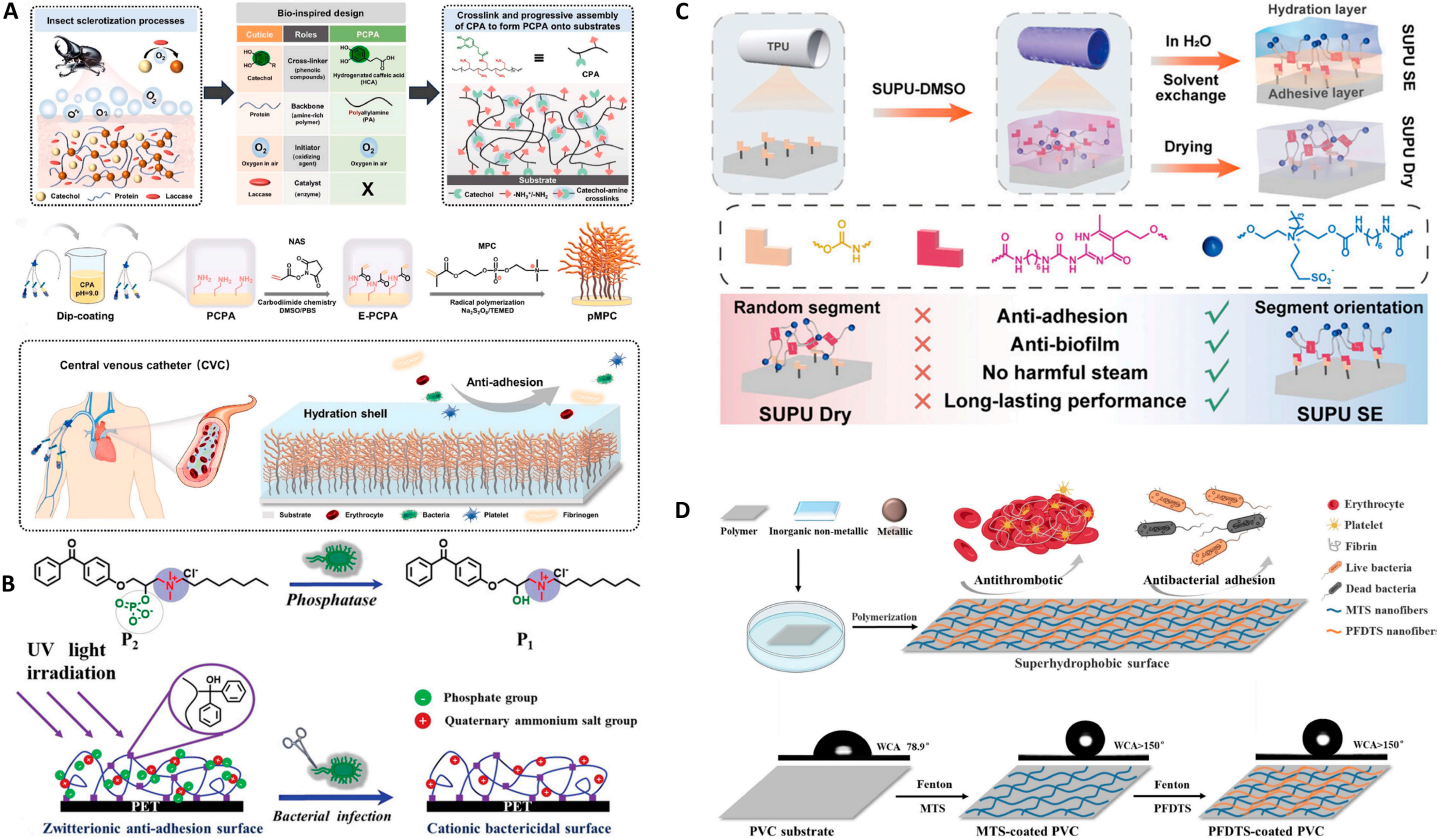
(A) Schematic diagram of developing pMPC armor by imitating the insect cuticle sclerotization process. Reprinted with permission [32]. (B) Schematic illustration of the alkaline phosphatase-responsive surface biomaterials [30]. (C) Mechanism of the SUPU SE and SUPU dry coatings, and excellent property of the SUPU SE coatings. Reprinted with permission [31]. (D) Schematic diagram of superhydrophobic coatings prepared with silane coupling agents after Fenton activation, possessing antithrombotic and antibacterial adhesion properties. Reprinted with permission [34].

The construction of a smooth surface in antifouling strategy can prevent the adhesion of bacteria and proteins on biomedical catheter. Inspired by the hard shell of insects, an anti-fouling coating of CVCs with excellent duration and effectiveness was reported [32]. The precoating phenol-polyamine film on catheters was formed by grafting poly-2-methacryloyloxyethyl phosphorylcholine (pMPC) molecular brushes via in situ radical polymerization (Fig. 2A). This superhydrophilic surface maintained good antibacterial and anticoagulant properties even after immersion in phosphate-buffered saline (PBS) for 30 d. Most zwitterionic polymer chains contain hydrophilic groups such as carboxyl, hydroxyl, and sulfonic acid groups, which have good hydration properties [51,52] and are widely used in the design of antibacterial adhesion and anti-thrombotic and anti-coagulant coatings. In addition, zwitterionic polymerization has a unique chain structure, and a pair of cationic and anionic groups is usually distributed on both sides of its structural unit molecules. They are electrically neutral at isostatic points, while the electrical properties of the polymer will alter to become a polyelectrolyte when the internal environment changes. Therefore, they have the potential to be developed into a controllable and adaptive antibacterial coating [53,54]. Based on this mechanism, a benzophenone-derived phosphatase light-triggered antibacterial coating was synthesized and tethered to the surface of a common catheter by light-triggered method [30] (Fig. 2B). Once alkaline phosphatase accumulated to a certain amount, the surface would immediately enter the bactericidal mode with high sensitivity and biocompatibility.

Catheters with complex shapes and narrow lumens are more likely to reduce the incidence of complications, but their application is limited by weak substrate adhesion and poor mechanical stability [55]. To address the complication, our group prepared a series of novel zwitterionic polyurethane (SUPU) with water-driven segment orientation by controlling the proportions of sulfobetaine-diol (SB-diol) and ureido-pyrimidinone (UPy) [31] (Fig. 2C). Under aqueous phase, the hydrophobic UPy groups gravitate toward the substrate, while the hydrophilic SB-diol groups move toward the surface, and there is a quadruple hydrogen-bonding motifs between the 2 groups. These are conducive to acquire robust stability and long-lasting anti-fouling. Among them, the best-performing group (SUPU3 SE) had excellent substrate adhesion and hydrophilicity [water-contact angles (WCAs) less than 10°], and achieved a 97.1% reducing protein fouling. Ren and colleagues [56] obtained a stable hydrophilic coating on the surface of silicone rubber by using tannic acid (TA) and 3-aminopropyltriethoxysilane (APTES), and modified the coating with Hep and poly(lysine) (PL) by layer-by-layer technique. The thromboplastin and thrombin time tests revealed distinguished extensions of 276% and 103%, and resistance to bacterial adhesion was increased by 99.9% compared to uncoated catheters.

Superhydrophobic coatings can also be used for antifouling. They possess remarkable properties of liquid repulsion and anti-adhesion. The mechanism is to construct a micro-/nanostructure on the surface of the substrate, in order that the forming air layer under the liquid reduces the contact area between the liquid and the substrate. Inspired by the superhydrophobicity of lotus leaves and the compound eye structure of mosquitoes, some scholars designed an indwelling catheter coating with micro-/nanohierarchical structures [57]. Such structures have optimal superhydrophobicity by reducing the adhesion site. Moreover, they can load drug well because of the porous and physical adsorptions. The experimental results show that the count of bacterial colonies in the membrane with added drugs decreased 1 to 2 orders of magnitude compared to the membrane without drugs, and even the membrane with the minimum concentration of 0.5 mg/ml reached over 96% of the antibacterial rate. The Fenton reaction has a strong oxidation ability. Functional groups such as hydroxyl and carboxyl groups can be introduced by this reaction. Wang and colleagues [34] used Fenton reaction and silane coupling to build a nanoscale topology structure with superhydrophobicity (Fig. 2D). The coating can be applied to a variety of substrates such as polymer, inorganic nonmetal, and metal. The property of superhydrophobicity was maintained even after immersion in PBS for 28 d.

### Active strategy for constructing antibacterial surfaces

The active strategies are to kill the bacteria. They work when passive strategies fail. There are usually methods of releasing antimicrobials and contact killing strategies. Researchers extracted and prepared silver nanoparticles to medical catheter coating surface, proving to have the function of the antibacterial and anti-biofilm formation [58]. Graft copper ions onto chitosan can endow anticoagulant and antibacterial functions on the catheter [59,60]. However, traditional methods of designing antimicrobial surfaces by coating metal nanostructures or mixing antibiotics inevitably face the problem of metal ions or antibiotic leakage, resulting in surface inactivation, toxicity, and antibiotic resistance [61–63]. Based on vitamin U motifs, our team prepared a sequence of alkylated sulfonium polypeptides through amino acid N-carboxyanhydrides (NCA) ring-open reaction. The alkylations and polymerization degrees of obtained polypeptides can be regulated, and among the reported antimicrobial peptides or peptoids, they exhibited an ultrahigh selectivity and anti-methicillin-resistant *Staphylococcus aureus* (*MRSA*) performances both in vivo and in vitro [64].

**Fig. 3.**
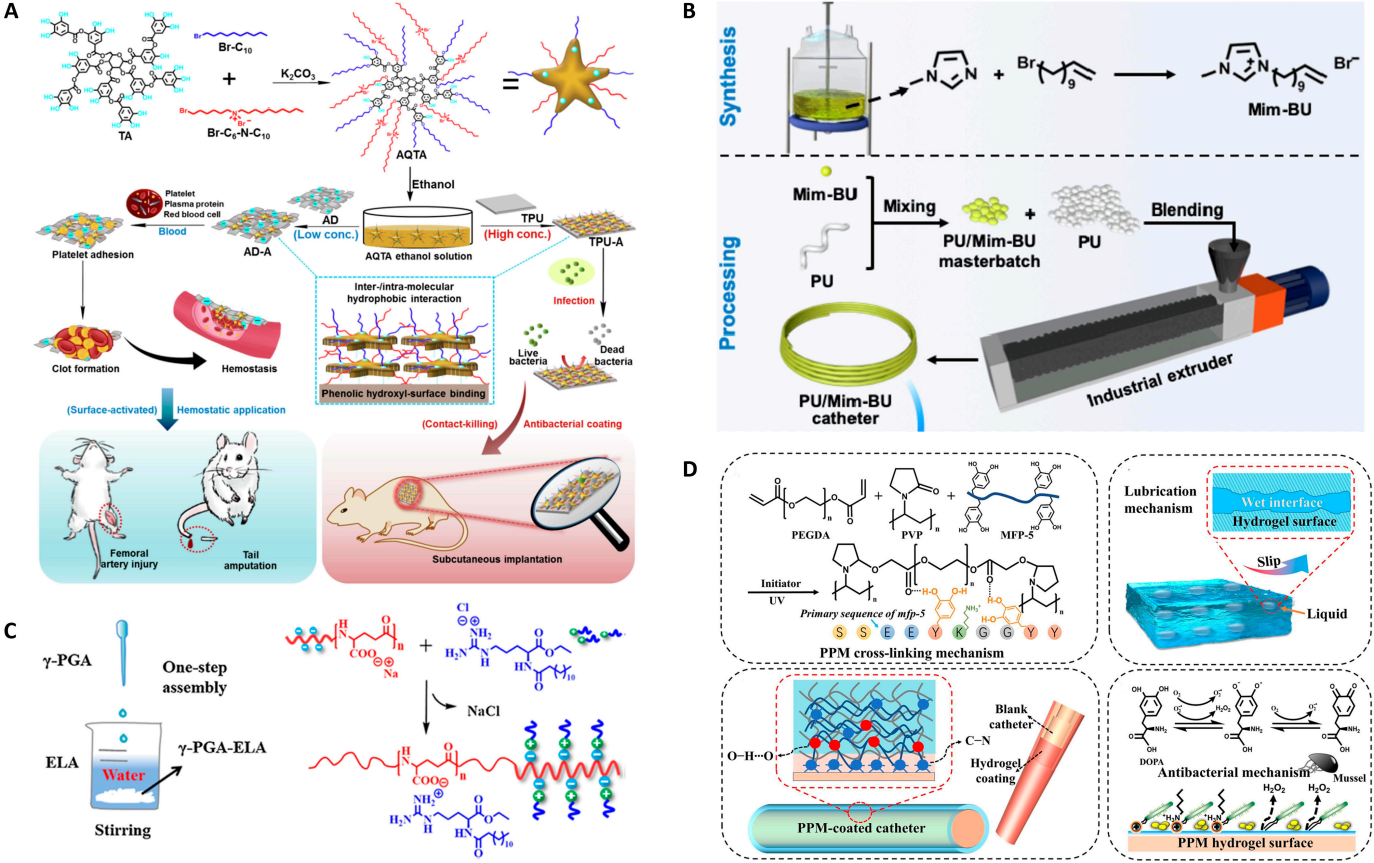
(A) Schematic diagram of the preparation of AQTA as well as the optimization of AQTA-coated TPU for antibacterial and hemostatic properties. Reprinted with permission [65]. (B) Schematic diagram of synthesis, preparation, and fabrication of the PU/Mim-BU catheters. Reprinted with permission [38]. (C) Schematic illustration of the preparation of the γ-PGA compound. Reprinted with permission [39]. (D) Schematic illustration of the crosslinking and the capacity mechanism of the PVP-PEG acrylate-coated catheter. Reprinted with permission [29].

In response to antibiotic resistance, a new strategy of cationic antimicrobial polymers has also been developed in recent years. This coating can act as resistant surface-anchored antimicrobials, but they are also often hemolytic active due to their membrane destruction behavior. A cationic coating composed of TA with alky and quaternary ammonium groups (AQTA) was designed [65] (Fig. 3A). Such coatings have antibacterial and hemostatic functions to show excellent hemostatic capacity on alginate dressing and gauze. In addition, the coating can promote coagulation of negatively charged dressings, but has a negative effect on the hemostatic performance of dressings with cationic chitosan.

Blending antibacterial agents with substrate is a common strategy for preparing antibacterial catheters. The antibacterial plastics were prepared by putting imidazole IL-typed cationic antibacterial agent into polyurethane (PU/Mim-BU). The mechanical properties are unaffected to maintain good antibacterial activity with minimum inhibitory concentration (MIC) values of 32 and 128 μg/ml for *S. aureus* and *Escherichia coli* under hydrodynamic flow and 3 times of autoclave sterilization treatments [38] (Fig. 3B). This method is simple to prepare and is expected to be industrialized. Some researchers used quaternary ammonium salt grafted on TA to synthesize quaternary tannic acid (QTA), and blended them with thermoplastic polyurethane (TPU) to prepare an antibacterial catheter, which had the best performance when the addition of QTA was 4% [66]. The percentages of viable bacteria above the surfaces of inserted specimens in vivo decreased from 90.13 ± 4.28 of TPU to 19.42 ± 5.04 of QTA-TPU in 3 d.

For more slender catheter coating, our group proposed a water-insoluble γ-PGA antibacterial compound with satisfactory hemocompatibility, histocompatibility, and low cytotoxicity via facile one-pot electrostatic assembly of γ-PGA and cationic ethyl lauroyl arginate [39] (Fig. 3C). The compound can easily form a colorless transparent coating on various substrates (inorganics, metal, and polymer), even on the lumen of slender catheters with 2 m of length and 1 mm of inner diameter. Experiments showed that the antibacterial effect of the lumen of catheters illustrated a log reduction of above 6 under dynamic flow condition.

Hydrogels have good hydrophilicity and flexibility, while such coating is easy to fall off the substrate because of their high swelling ratio and wettability. In this case, improving the substrate adhesion of hydrogel antibacterial coating is necessary. Based on characteristics of biomimetic adhesion and antibacterial coating of recombinant mussel protein (MFP) [67,68], Wang and colleagues [29] constructed a double network hydrogel coating with chemically crosslinked network of PVP-PEG acrylate and physical network of MFP-5 (Fig. 3D). The formed network still maintains excellent antibacterial property and super-lubricating capacity under 100 consecutive frictions, with a minimum antibacterial concentration of 500 μg/ml and a friction coefficient of less than 0.05.

Biomimetic rough nanoparticles (RNPs) can adhere to bacteria and then kill them more effectively. However, the bare silica is inert. It is necessary to load the antibacterial agent. An RNP with template of an antibacterial drug of benzalkonium chloride was synthesized in situ to enhance antimicrobial properties compared to their counterparts [69]. Additionally, the composite materials can be applied to fabrics and still has antibacterial ability after 5 times of washing. Many natural substances can be used to resist bacteria directly. Some composite materials prepared by mixing Chinese herbal extracts (mugwort, honeysuckle, etc.) and natural rubber latex had been proven to have good antibacterial, biocompatibility, and mechanical properties [70].

### Active and passive combination strategy for constructing antibacterial surfaces

**Fig. 4.**
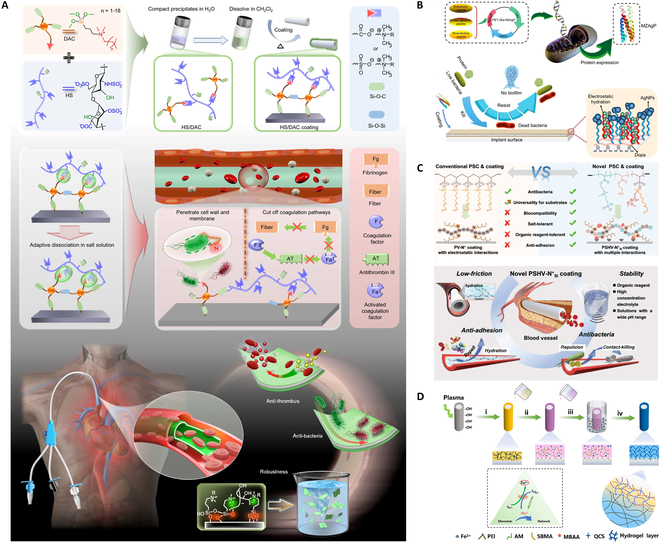
(A) Schematic illustration of the preparation, dissociation properties, and multifunctionality of heparin sodium/organosilicon quaternary ammonium surfactant coating. Reprinted with permission [71]. (B) Schematic diagram of preparation of MZAgP coating as well as its anti-adhesion and antibacterial properties. Reprinted with permission [73]. (C) Schematic illustration of exceptional capacities of the current novel polyelectrolyte–surfactant composite (PSC) coatings such as antibacterial, anti-adhesion, low-friction, and stability. Reprinted with permission [81]. (D) Schematic diagram of the preparation process of the hydrogel coating. Reprinted with permission [82].

The combination of both active and passive strategies is firmed to be a more ideal antibacterial treatment to prevent early adhesion and kill bacteria. Lately, we had synthesized a multifunctional coating complex composed of Hep sodium and quaternary ammonium silicone surfactant, which can be attached to any shape of medical devices through a simple impregnation process [71] (Fig. 4A). The coating with adaptive antithrombosis and antibacterial infection not only shows good stability under extreme conditions but also significantly reduces thrombosis adhesion by 60%. In vitro and in vivo experiments showed a broad spectrum of antimicrobial activity (>97%). This coating could meet the demand of fighting catheter-associated bloodstream infections and clots in the clinic. To deal with bacterial infections and multiple encrustations, we designed a salt-triggered chondroitin sulfate complex coating, which can be applied in urological devices with arbitrary shapes. The complex coating exhibits potency to prevent urological medical device-related complications with significant reduction of calcium content of more than 90% [72].

A triblock protein (MZAgP) consisting of zwitterionic peptide, mussel-adhesive peptide, and silver-binding peptide was obtained to show a “kill-releasing” and antibiofilm performance [73] (Fig. 4B). The coated catheters maintained bacteriostatic capacities throughout 168 h under hydrodynamic circumstances, and also showed superb antifouling and antibacterial properties even stored for 2 months [74]. Chen and colleagues [75] endowed PU antibacterial and antifouling surface by using CuSO_4_/H_2_O_2_ to make polydopamine and poly-(sulfobetaine methacrylate) co-deposition. The zwitterions in the coating improved surface wettability significantly, reducing protein adsorption. In addition, the metal-phenolic networks released copper ions, entitling them over 90% antibacterial performance against *E. coli* and *S. aureus* markedly.

The AgNPs-PAAm-CS-PVP hydrogel had a more durable release of the antibacterial agents of AgNPs and Ag+, making the coated urinary catheters to have long-term antibacterial activity [76]. Mixing polydopamine, silver NPs, and methylene can prepare the composite coating with a low release rate of silver ions, and the release of silver ions is 16.95% of the total amount within 14 d [77]. A mixture of dimethyl siloxane, metallic silver, and zinc powder can be prepared, which inhibits biofilm and growth of *E. coli* more than 6 d [78]. Our group prepared an antifouling antibacterial coating with fluoride block copolymer, and the minimum bacteriostatic concentration (MIC) against *E. coli* and *S. aureus* was calculated to be 3.91 and 1.95 μg/ml, respectively [79]. In addition, they can emit strong blue light under the irradiation of 350 nm, providing a method for detecting the uniformity of the inner wall of the coated catheters.

Zhang and colleagues [80] constructed multifunctional medical composite amphiphilic nanofibrillated cellulose (Am-CNF) with inherent antimicrobial and antifouling properties and self-healing ionic polyurethanes (HPU). Our group developed a multifunctional polyelectrolyte–surfactant composite coating using organosilicon quaternary ammonium surfactant (N^+^_Si_) [81] (Fig. 4C). The coated catheter expresses great antibacterial activities in vitro and in vivo while improving cell survival by 87% and reducing protein adhesion more than 70%. The formation of biofilm is reduced by about 99.6%. The complex can rapidly assemble in one step through hydrophobic and electrostatic interactions between N^+^_Si_ and adjustable polyelectrolyte with properties of crosslinkable, anti-adhesive, and anionic groups. Benefiting from the crosslinking structures, the biocompatibility and resistance to various bacterial adhesion were improved.

Gao and colleagues [82] use polyethyleneimine (PEI) as adhesive layer to combine matrix and hydrogel coating by covalent bond (Fig. 4D). The hydrogel coating is prepared by polymerizing acrylamide (AAm) and sulfobetaine methacrylate by redox-based crosslinking method in the presence of quaternary ammonium chitosan (QCS). It showed low friction coefficient, good antibacterial properties (95.88% and 97.11% inhibition rates to *E. coli* and *Bacillus subtilis*, respectively), and anti-nonspecific protein adsorption to inhibit the platelet adhesion for thrombosis (74.7 s).

Zheng and colleagues [83] synthesized a quaternary ammonium cationic ligand successfully, improving the reunion of the titanium dioxide nanoparticles and its antibacterial property dependence of light. Feng and colleagues [84] designed and synthesized a kind of anti-fouling and “defensive” hydrogel coating. When confronted with bacterial infections, coating can release gentamycin sulfate (GS) to kill bacteria. The infected coating is stripped to renew the stain-resistant surface. QCS derivatives were synthesized to modify the surface of TPU. Compared with the bare TPU surface, the antibacterial activity was maintained for 8 weeks after immersion in serum, and the bacteria in the surrounding tissue were reduced by 99.87% after 5 d [85]. Hydrogels containing AAm, 2-methylacryloxyethylphosphocholine (MPC), and zinc methacrylate (ZMA) have been shown to have enhanced antimicrobial and stain resistance, with water contact angles reduced from 107.01° to 23.22° [86]. The deposition of bovine serum albumin (BSA) on silicone rubber surface decreased from 0.33 to 0.07 mg/cm^2^.

### Stimulus response strategy for constructing antibacterial surfaces

**Fig. 5.**
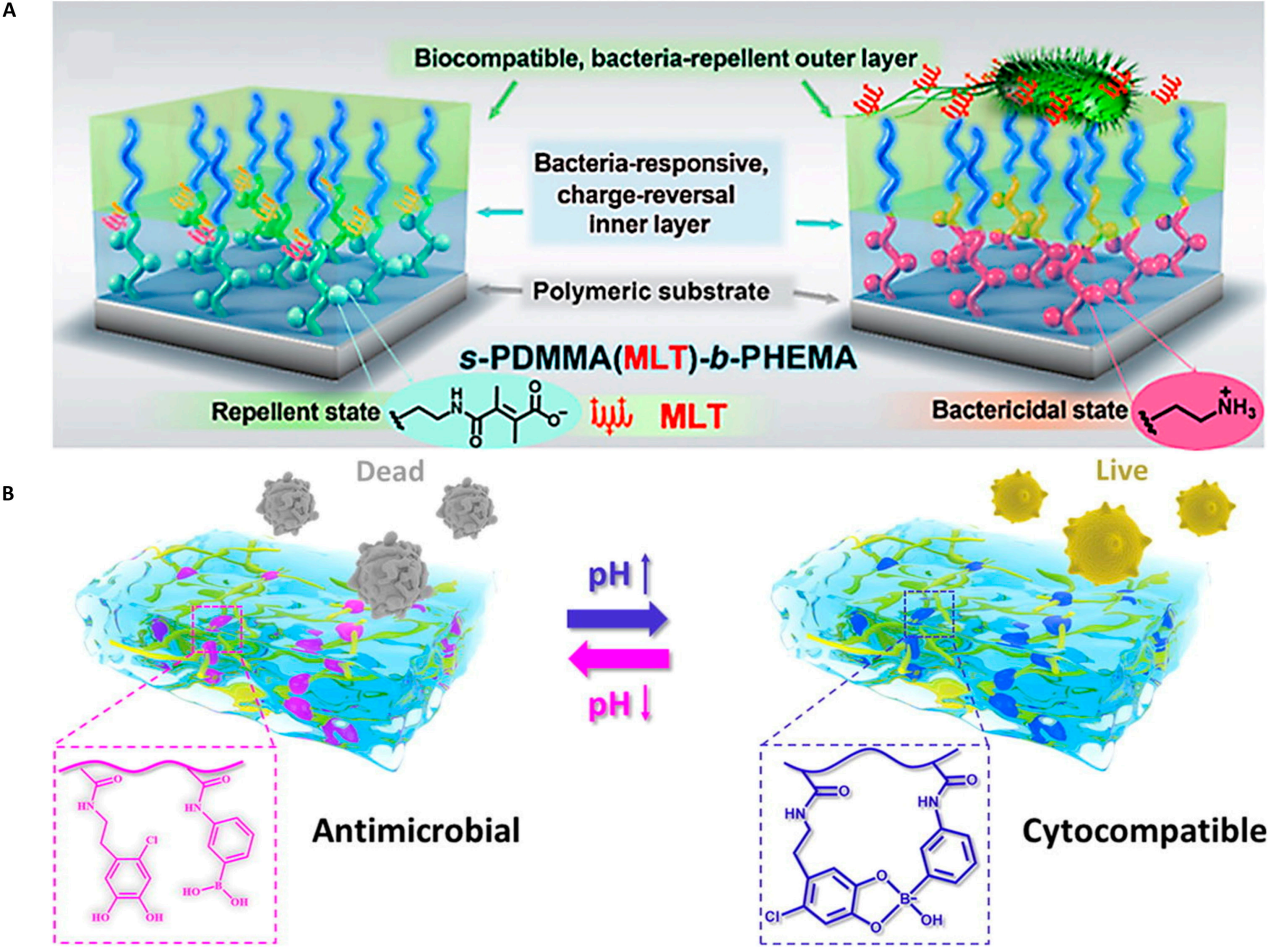
(A) Schematic drawing of the hierarchical polymer brush coating with biocompatible and nonadhesive performance. Reprinted with permission [88]. (B) Mechanism diagram of pH-responsive antibacterial hydrogel based on catechol–boronate complexation chemistry. Reprinted with permission [89].

Monitoring the microenvironment caused by bacterial infection can improve the effectiveness of antibacterial coating and prevent early infection. Bacterial infections can lead to acidic microenvironment [87], and this feature can be utilized to construct pH response coating. Our group reported a polymer brush that is applicable to inorganic antibacterial materials [88]. In the hierarchical structure of polymers, poly(hydroxyethyl methacrylate) (PHEMA) acts as the hydrated outer layer, preventing the initial adhesion of bacteria under neutral conditions (Fig. 5A). Once existing bacterial infection, the amide bond in the inner layer can releases the loaded melittin (MLT) to kill the adsorbed bacteria in time. The number of planktonic bacteria was reduced over 98% compared to monolayer structures. Zhang and colleagues [36] synthesized a hydrogel coating with water-responsive Janus adhesion and acidity-triggered transformation, exhibiting 44.6 kPa of substrate adhesion strength and 0.03 of friction coefficient of superlubricity. Such a hydrogel coating has also been shown to enable dynamic regulation of the antimicrobial microenvironment through adaptive release of chitosan/BSA-gold nanoparticles and silver nanoparticles.

A hydrogel with the pH-responsive antibacterial activity is put forward on the compound of chlorinated catechol and phenylboronic acid [89] (Fig. 5B). Under the acid buffer, the mechanism is that the complexes dissociate, exposing the toxic catechol chloride to play a bactericidal role. In the alkaline buffer, the catechol and borate form a complex to reduce cytotoxicity effectively [90–92]. The hydrogel was proved to have excellent antimicrobial capacity against multiple strains, for example, the colony-forming units (CFU)/ml of *MRSA* reached reduction of 4 log_10_ from 10^8^ in a pH-responsive antibacterial activity and lower cytotoxicity.

**Fig. 6.**
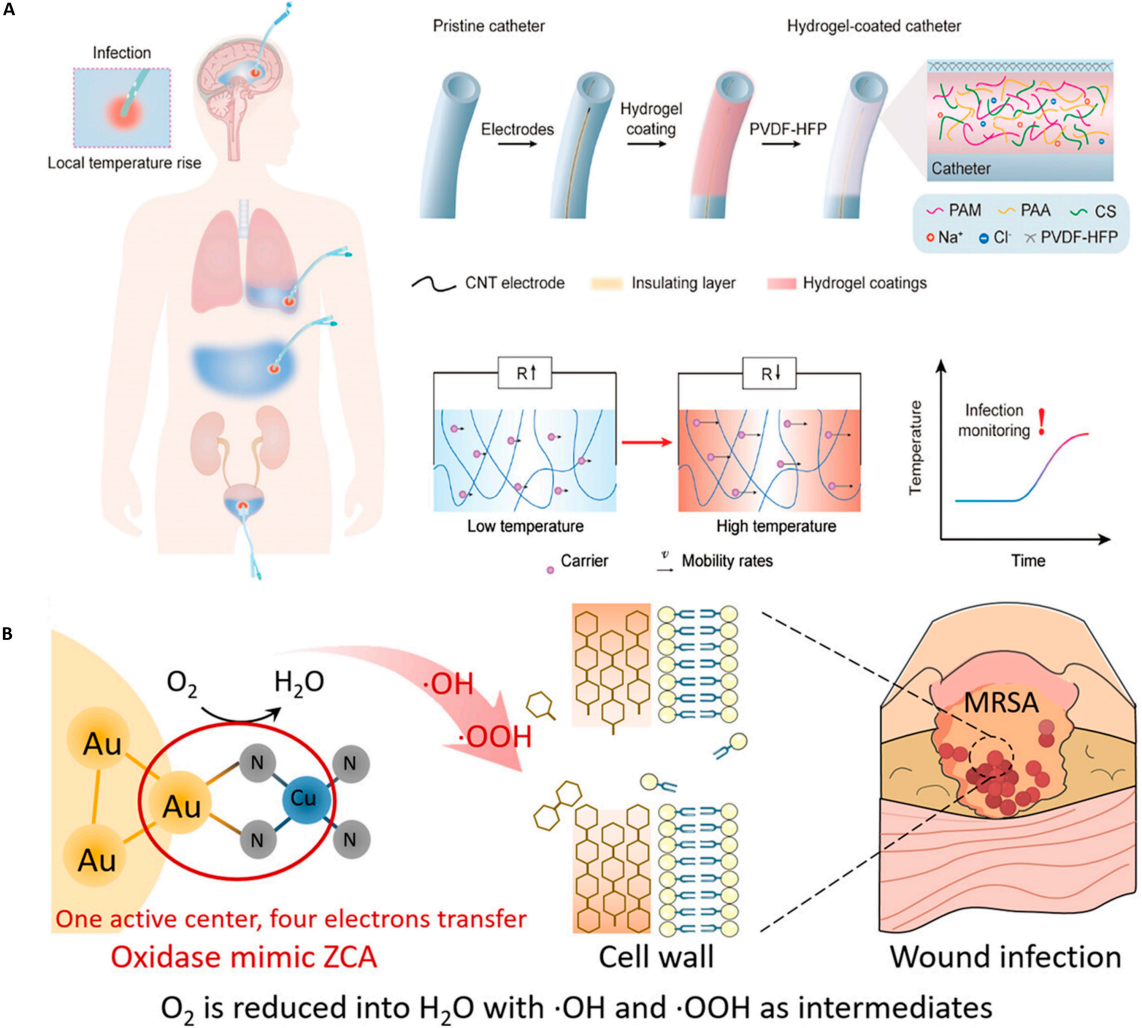
(A) Schematic diagram of the temperature-sensing hydrogel coating applied to medical catheter. Reprinted with permission [94]. (B) Schematic illustration of Au–N–Cu active center and further combat MRSA [117].

Bacterial infections can cause local temperature changes [93]. Therefore, Zhang and colleagues [94] synthesized a real-time temperature-sensing hydrogel coating via in situ polymerization (Fig. 6A). The structure consists of carbon nanotube fibers that act as electrodes for sensing and a stable poly(AAm/AA/chitosan) triple network. The mechanism of monitoring is that Na^+^ and Cl^–^ in the hydrogel form a stable ion flow whose rate increases as the hydrogel’s temperature increases, under the alternating electric field. In this way, we can convert the change of temperature into an electrical signal to be detected. The hydrogel coating had reached a temperature resistance coefficient of 2.90% °C^−1^ and Young’s modulus of 4.24 kPa. Under the brain infection model, the individual survival rates reached 90% by providing early warning of the sensor.

Besides pH, temperature changes, and specific overexpression enzymes, some natural responses caused by the immune system, for example, oxidative stress, can also be used as a marker to develop stimulus response coating [95–100]. In recent years, PDT has been widely studied as a promising antimicrobial strategy that does not trigger bacterial resistance and has a broad spectrum of antibacterial activity. ROS can destroy active substances in bacterial cells and trigger oxidative stress [101,102]. Electron transfer (ET) will stimulate the generation of ROS [95]. Some semiconductor nanoparticles were shown to promote this process. Aggregation-induced emission luminogens (AIEgens) [103–105] can produce ROS to construct antibacterial coating in the visible light. Tang and colleagues [106] prepared a remarkable antibacterial coating with 2 types of AIEgen with positive charges as photosensitizer molecules. Negatively charged chemical groups can be introduced into the surface of a variety of medical devices managed by oxygen plasma technology. As a result, the AIEgen with positive charge can be easily and tightly assembled on the surface. The AIEgen-based coatings could effectively resist 8 kinds of normal and multidrug-resistant bacteria including pernicious MRSA in a short period. Moreover, the high biocompatibility and low inflammation incidence were proved by assays in vitro and in vivo.

Yan and colleagues [107] embedded carbon quantum dots (CQDs) and MoS_2_ QDs in dendritic fiber nanosilicon dioxide, preparing light response antibacterial coating, and the bacteriostatic activity of common bacteria was above 95%. Sotiriou and colleagues [108] prepared visible light catalytic activity of silver/titanium oxide nanoparticles and deposited them on the substrate as a porous coating. Next, the nanocomposite coating was prepared by injecting the polymer into it with the function of multiple triggering and no cytotoxicity. Our group prepared an electrospun nanofiber mat with biocompatible and antimicrobial performances by co-electrospinning of polyanionic poly (γ-PGA) and cationic photosensitizer 5,10,15,20-tetrakis(1-methylpyridinium-4-yl) porphyrin tetra (p-toluenesulfonate) (TMPyP) in aqueous solution, and then enhanced stability by the chemical crosslinking [109]. The antibacterial assay in vitro demonstrated that they can effectively kill the broad-spectrum bacteria even with a relatively low loading dosage of TMPyP (e.g., 0.1 wt%). Moreover, those nanofibrous mats restrained inflammatory reaction and also exhibited negligible local toxicities, benefiting to the wound healing.

Therapeutic platforms with enzyme-sensitive and light-activated carbon monoxide releasing molecules may provide possibility for the detection and elimination of early bacterial infections [110]. Combining the red emission of carbon nanotubes with sensitive to near infrared (NIR) into a polymer matrix can construct a photothermal antibacterial agent with automatic tracking behavior [111]. Our team designed molecular fluorescent nanoprobes (HF-D-Ala NPs) via facile self-assembly. When encountering bacteria, HF-D-Ala NPs are disassembled, and then the free HF-D-Ala can be integrated into cell walls. When encountering bacteria, HF-D-Ala NPs are disassembled, and then the free HF-D-Ala can be integrated into cell walls to realize visual bacterial detection and imaging. Subsequently, the marked bacteria can be killed by carbon monoxide released from embedded HF-D-Ala upon visible light [112].

PTT can eliminate pathogenic cells by breaking metabolic signals and membrane permeability, denaturing proteins/enzymes, and making bacterial death. Based on PTT, Xu and colleagues [113] prepared one TA-reduced gold nanoparticles (Au@TA NPs)/PEG layer with antifouling and antibacterial functions under the NIR irradiation. Because of the photothermal conversion of Au NPs and antifouling property of PEG, the polydimethylsiloxane (PDMS)-TA-PEG-Au surface shows an excellent bactericidal effect in vitro and in vivo, for example, resistance to *E. coli* and *S. aureus* above 99%.

The metal–organic frameworks (MOFs) consisting of metal ions and organic ligands could serve as carriers for various substances [114,115]. Researchers have prepared a coating with a self-activated 3-dimensional periodic MOF structure that can release nitric oxide free radicals from endogenous donor—S-nitrosoglutathione (GSNO), making the anti-fouling surface. The assay shows that bacteria cells are almost absent in the presence of GSNO, giving up to 10^7^ CFU of bacteria in the surrounding suspension [116]. Further, our team reported a new ET way in the MOF-based oxidase mimics. We constructed a unique active center of Au–N–Cu domain between Au nanoclusters (NCs) and CuN_4_ single sites to enhance ROS against drug-resistant bacteria. The oxygen reduction was catalyzed by a direct 4-ET process without specific substrates. The oxidase-like material reduced 4.7 logs MRSA number in 3 h [117] (Fig. 6B).

However, most ROS agents are dependent on lighting conditions, and their clinical applications under no light conditions or deep bacterial infection sites is limited. Zeolite imidazolate framework (ZIF) is an ideal oxygen-activated material with easy biodegradation, large surface area, and easy oxygen absorption. Recently, Chen and colleagues [118] reported a kind of ZIF materials doped by Cu atoms that can generate ROS under dark conditions. In addition, ultrasound can also be used as an external stimulus to trigger ROS antibacterial processes. Yang and colleagues [119] prepared an antimicrobic tube coated with a polyvinylidene difluoride-hexafluoropropylene (PVDF-HFP) membrane with piezoelectric zinc oxide (ZnO) NPs. In vitro, the ZnO-PVDF-HFP coating restrains bacterial growth and formation of biofilm under ultrasound-mediated mechanical stimulation up to 4 weeks. More importantly, the switch and strength of antimicrobic activity could be adaptively adjusted via ultrasound.

### Other strategies for constructing antibacterial surfaces

**Fig. 7.**
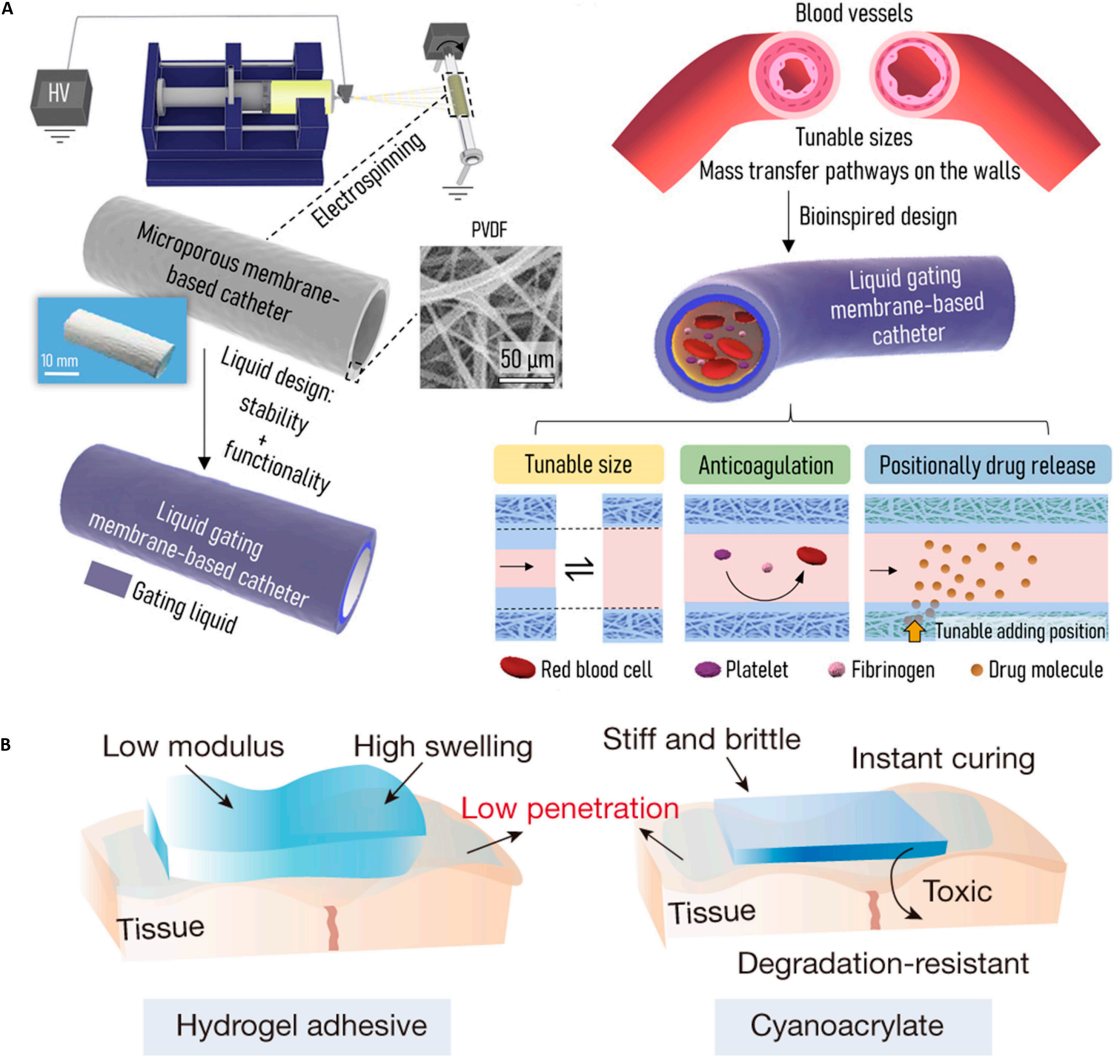
(A) Schematic diagram of designing and preparing of bioinspired LGMC. Reprinted with permission [122]. (B) Schematic views of the adhesion of hydrogel adhesives [123].

In addition to the abovementioned strategies of building antibacterial coatings directly, several methods of improving the properties of coating have also been reported. A new strategy was reported to protect medical tubing antibacterial film. Formed coating with C/SiO_2_ protective layer can resist to dissolve 14 d in human urine [120]. A self-adaptive liquid gating membrane-based tube [121] was prepared with properties of anticoagulation and positionally drug release [122]. The experiments show that a few clots are left on liquid gating membrane-based catheter (LGMC) after 0.5 h of whole-blood flow. The catheter expresses function of adaptive tube size control by the stable interfacial structure of the affinity between the gating liquid and the porous film. Moreover, the gating liquid can be dynamically distributed inside the porous membrane on both sides of the tube wall. This not only endows biocompatibility properties for catheter applications but also extends the specific fluid-based mass transfer pathways on catheter walls (Fig. 7A). We utilized precursor of hydrophilic acrylate and hydrophobic cyclic ketene acetal monomer for designing a backbone-degradable robust adhesive (BDRA) with robust adhesion, good biocompatibility, and degradability via redox-initiated in situ ring-opening polymerization. It is worth noting that the BDRAs exhibit high mechanical modulus (100 kPa to 10 GPa) and more excellent adhesion strength on various tissues, wet bone >16 MPa and porcine skin >150 kPa, compared to that of commercial cyanoacrylate superglue of about 4 MPa and 56 kPa [123] (Fig. 7B).

## Conclusion and Prospects

Thrombus and bloodstream infections caused by the bacterial adhesion and biofilm formation on implant catheters are serious clinical problems that have not yet been addressed. In this review, we focus on recent advances based on the different mechanisms of antimicrobial polymer coatings to show great potential in preventing bacterial adhesion and colonization (Table). Passive antibacterial coatings can form a physical barrier (such as a hydration layer) against initial bacterial attachment; however, the antibacterial action lacks effectiveness and long-term nature. Active antibacterial coating is a hotspot to improve the surface bactericidal activity, but this method may be invalidated due to the protein adhesion. The combination of active and passive features into one coating is to optimize the polymer coating to enhance surface antibacterial properties. The point is to minimize interferences between different functional components. In response to the threat of multidrug-resistant bacteria, stimulus-responsive antibacterial coatings often provide more specialized antibacterial effects. In addition, early monitoring and tracking of bacterial infections is more conducive to treatment. At present, many non-antibiotic strategies have been widely studied for their safer and more effective characteristics, such as PDT, chemodynamic therapy, PTT, and sonodynamic therapy. The synergistic effects can overcome the limitations of monotherapy to kill bacteria more easily.

**Table.** The main types of surface modification strategies used in implant catheters

Catheter type	Name	Constitute	Application scenarios	Antibacterial effect	Characteristic	References
Active strategy	Hydrogel coating (PPM)	PVP-polyethylene glycol acrylate and recombinant mussel protein	Urinary catheter	Minimum antibacterial concentration: 500 μg/ml, friction coefficient less than 0.5	Antibacterial property and super-lubricating	[29]
	PU/Mim-BU	PU and imidazole IL-typed cationic antibacterial agent	Blending antibacterial agents with substrate	MIC values of *S. aureus* and *E. coli* are 32 and 128 μg/ml	Efficient and stable antibacterial performances without negative effect to the mechanical properties	[38]
Passive strategy	PMPC	Phenol-polyamine film and poly-2-methacryloyloxyethyl phosphorylcholine (pMPC)	Central venous catheters (CVCs)	Long-term antibacterial and anticoagulant in PBS for 30 d	Anti-fouling, antibacterial, and anticoagulant	[32]
	SUPU3 SE	Sulfobetaine-diol (SB-diol) and ureido-pyrimidinone (UPy)	Catheter with complex shapes	WCAs less than 10°, 97.1% of reducing protein fouling	Long-lasting anti-biofouling properties, excellent substrate adhesion	[31]
Active and passive combination strategy	Heparin network-mediated coatings	Hep sodium and quaternary ammonium silicone surfactant	Any shape of intravascular catheters	Broad spectrum of antimicrobial activity (>97%)	Adaptive antithrombosis and antibacterial infection	[71]
	A triblock protein (MZAgP)	Zwitterionic peptide, mussel- adhesive peptide, and silver-binding peptide	Antifouling and antibacterial catheters	Long-term antibacterial for 168 h under hydrodynamic circumstances	“Kill-releasing” and antibiofilm performance	[73]
	Hydrogel coating	Acrylamide (AAm), sulfobetaine methacrylate, quaternary ammonium chitosan (QCS)	Medical catheters	95.88% and 97.11% inhibition rates to *E. coli* and *B. subtilis*	Excellent anti-fouling and anti-thrombosis	[82]
	Polyelectrolyte-surfactant composite coating	Organosilicon quaternary ammonium surfactant and polyelectrolyte	Medical catheters	Cell survival by 87%, anti-adhesion 70%. Anti- biofilm 99.6%	Biocompatibility and resistance to various bacterial adhesion	[81]
Stimulus response strategy	Au@TA NPs/PEG (TA-PEG-Au) layer	PEG, gold nanoparticles, and polydimethylsiloxane (PDMS)	Medical devices	Resistance to *E. coli* and *S. aureus* above 99%	Photothermal, antifouling, and antibacterial	[113]
	ZnO-PVDF-HFP membrane	Piezoelectric zinc oxide NPs (ZnO NPs), polyvinylidene difluoride-hexafluoropropylene	Silicone catheters	Anti-biofilm under ultrasound up to 4 weeks	Applicable to inorganic antibacterial materials, restrain bacterial growth and formation of biofilm	[119]
Other strategies	Backbone-degradable robust adhesive (BDRA)	Hydrophilic acrylate and hydrophobic cyclic ketene acetal monomer	Adhesive on various tissues	Mechanical modulus (100 kPa to 10 GPa), adhesion strength on porcine skin >150 kPa, wet bone >16 MPa	Robust adhesion, biocompatibility and degradability	[123]
	Liquid gating membrane-based catheter (LGMC)	Liquid and porous membrane	Catheter	Few clots left on LGMC after 0.5 h of whole-blood flow	Anticoagulation and positionally drug release	[122]

These abovementioned antimicrobial coating shows a potential application in medical catheters. However, there are still several challenges to be addressed in the clinic, including long-term antibacterial efficiency, low manufacturing costs, simple and practical production processes, and biosafety. (a) Further strengthen the monitoring and diagnosis of early bacterial infection: For example, if bacterial and fungal infections occur in the heart and ventricular drains, the body response is delayed usually and may lead to increased mortality. (b) Understanding the structure–performance relationship against bacterial adhesion and biofilm formation: This can help us to develop the coating with new bactericidal mechanism and more targeted biomedical catheters suitable for different scenarios to improve the antibacterial function effectively. (c) Conducting in vivo experiments and preclinical studies to achieve clinical conversion of antibacterial polymer coatings: Research and optimize preventive measures in deep, such as the development of new antibacterial coatings (biodegradable, biocompatible) and optimization of medical materials.
